# Analysing outlier communities to child birth weight outcomes in Malawi: application of multinomial logistic regression model diagnostics

**DOI:** 10.1186/s12887-022-03742-z

**Published:** 2022-11-26

**Authors:** Natasha Sakala, Tsirizani M. Kaombe

**Affiliations:** Department of Mathematical Sciences, School of Natural and Applied Sciences, University of Malawi, Zomba, Malawi

**Keywords:** Child birth weight, Multinomial logistic regression, Diagnostic statistics, Outlier communities

## Abstract

Studies have reported significant effect of geographically shared variables on new-born baby weight. Although there is growing use of community-based child health interventions in public health research, such as through provinces, schools, or health facilities, there has been less interest by researchers to study outlying communities to child birth weight outcomes. We apply multinomial logistic regression model diagnostics to identify outlier communities to child birth weight in Malawi. We use a random sample of 850 clusters, each with at least 7 households based on 2015-16 Malawi demographic and health survey data. There were a total of 11,680 children with measured birth weight, that was categorised as either low ($< 2,500$ grams), normal ($2,500 - 4,000$ grams) or high ($> 4,000$ grams). The analyses were done in STATA version 15 and R version 3.6.3. Based on a multinomial logit model with various socio-demographic factors associated with child birth weight, the results showed that two clusters from rural parts of Southern region of Malawi had overly influence on estimated effects of the factors on birth weight. Both clusters had normal to high birth weight babies, with no child having low birth weight. There could be some desired motherhood practices applied by mothers in the two rural clusters worth learning from by policy makers in the child healthcare sector.

## Introduction

Birth weight is the weight of the baby at birth, measured in grams or kilograms. This has implications on the baby’s future morbidity and mortality outcomes. For the baby’s health assessment’s sake, the child birth weight is categorised into three groups in accordance with World Health Organisation (WHO) standard: low birth weight ($< 2,500$ grams), normal birth weight ($2,500-4,000$ grams), and high birth weight ($> 4,000$ grams). The risk of a baby being born with low birth weight is high in sub-Saharan African region [1]. Several factors have been reported in literature as having strong association with low birth weight in children, some genetic [2] and others socio-economic [3–5].

There is also evidence in literature that the risk factors of low birth weight are shared by children over a geographical area, mostly from studies that analysed spatial patterns of birth weight at wider geograpical units, such as districts or provinces [6–8]. The health outcomes of children who are studied over a wide geographic area may have weaker within-group correlations compared those studied in short-spaced locations, such as within communities, schools, or villages, since the known and unknown variables that influence the health outcomes are shared by the children who stay closeby. However, there is limited research on outlier communities to birth weight outcomes in sub-Saharan African countries. This article applies diagnostic statistics for multinomial logistic regression model to identify outlying clusters to birth weight outcomes in Malawi using the 2015-16 Malawi demographic and health survey data.

An outlier community to child birth weight means a group of children staying in some neighbourhood, whose birth weight measurements deviate markedly from those of the children that live in the other distant communities [9]. Identifying the outlier community to child birth weight may help in understanding unique factors that precipitate the observed pattern of birth weight in that community. For instance, predominant low birth weight in some community may relate to exposure to some environmental risk factors by expectant mothers of that community, such as air pollution [10] and exposure to unfamiliar cultural practices by mothers of the community, such as inapproriate antenatal care [11] among others. Although there is growing interest by public health researchers to study and implement community-based child health interventions, for example using schools, hospitals, and villages as units of analysis [12–15], the statistical techniques for flagging outlier groups of observations are seldom applied to support such analyses. This problem is much common in studies that apply nonlinear regression models, such as multinomial logistic regression, where diagnostic statistics for grouped data are not much developed compared to linear regression models [16, 17]. Hence, this study demonstrates a unique contribution to the application of nonlinear regression model diagnostic statistics in detecting unusual grouped birth weight patterns in a sub-Saharan Africa setting.

Knowledge of the communities that have unusual birth weight outcomes compared to the rest will help relevant policy makers in maternal and child health sector to formulate targetted interventions for improving the child health in the affected communities. This section is followed by a section on methods. Thereafter, the results are presented in Section 3, followed by the discussion and conclusion in Sections 4 and 5, respectively.

## Methods

Secondary child data from the “kids’ records” file in the 2015-16 Malawi Demographical Health Survey (MDHS) were analysed in this study. The survey engaged two-stage stratified sampling, where 850 emuneration areas (clusters) were randomly sampled from across the country at first stage and 27,516 households were sampled from these clusters at second stage using rural and urban stratification [18]. The households had 13,448 children whose mothers or adult caregivers had responded to questions related to child birth weight, of whom 11,680 had verifiable birth weight values, while 1,768 had missing birth weights, and were therefore dropped from analysis, as the ultimate sample was large enough for the intended analyses in this study. The data are freely available for users at www.DHSprogram.com.

During analysis, the outcome variable - birth weight was split into three categories, based on WHO standards as indicated in Section 1, namely: low birth weight - LBW ($< 2,500$ grams); normal birth weight - NBW ($2,500-4,000$ grams); and high birth weight - HBW ($> 4,000$ grams) [1]. The analyses used explanatory variables that were reported useful for predicting child birth weight in previous studies, and these included mother’s age at child birth, household wealth quintile, child birth order, mother’s education level, place of residence, whether the birth was singleton or not, sex of child, frequency of antinatal care visits, mother’s smoking status during pregnancy, and whether the birth was through caesarian section was not [4, 19–21]. Other factors reported to be associated with child birth weight include mother’s weight, mother’s height, and mother’s body mass index (BMI) [20], however these had a lot of missing values in the 2015-16 MDHS, hence they were not included in the analysis. The model estimates were computed using STATA version 15.0., while all figures were processed using R version 3.6.3 software.

A survey weighted multinomial logit regression model was used to analyse the data because the reponse variable, birth weight, had three levels as highlighted in the previous paragraph and was assumed to follow a multinomial probability distribution [22]. Let $y_{ij}$ be a binary outcome with a value of 1 if *i*-th child’s birth weight falls in category *j* $(i = 1, 2, ..., n_{j}; j = 1, 2, 3)$ and 0 otherwise, and let $w_i=\frac{N_c}{n_c}$ be the sampling weight for the *i*-th child in a particular survey cluster *c* and one of the two strata, where $N_c$ is the population of under-five children in cluster *c* and $n_c$ the sampled children in the cluster. Further, let ${\textbf {x}}^{T}_{ik}=(1,x_{i1},x_{i2},...,x_{ip})$ be a vector of observed covariates for the *i*-th child, $(k = 1,2,...,p)$. In addition, let each observation $y_{ij}$ have a distinct conditional survey weighted probability of belonging to *j*-th category given the covariates as:

1 $$\begin{aligned} \pi _{j}(\mathbf {x})=P(Y=j|\mathbf {x})=\frac{exp(\beta ^T_j\mathbf {x}w_i)}{\sum \nolimits _{s=1}^{j}exp(\beta ^T_s \mathbf {x}w_i)} \end{aligned}$$

where $\beta ^T_j=(\beta _{0j},...,\beta _{pj})^T$, for $j=1,2, 3$. In this case, $\pi _{3}(\mathbf {x})=\frac{1}{\sum \limits _{s=1}^{j}exp(\beta ^T_s\mathbf {x}w_i)}$ because category 3 acts as a baseline in the estimation process [17, 22, 23]. To compute $w_i$, the population size for the clusters, $N_c$ was estimated using data from 2018 national census, which was closest to the 2015-16 MDHS that was used, while $n_c$ was given per each cluster in the survey data. The rural and urban stratification was used for strata weights during model estimation, and the *svy* command was used in Stata version 15 to define the sampling weights. There were 2, 645, 948 children under the age of five years in Malawi as of 2018, this, along with the DHS sample size of 11, 680, was used to compute the finite population correction (fpc) factor, $\sqrt{\frac{N-n}{N-1}}=\sqrt{\frac{2,645,948-11,680}{2,645,948-1}}=0.9978$, which was close to 1, and hence it was ignored in the survey weighted estimates of model coefficients [23, 24].

Then, for the 13,448 independently observed children, $\sum y_{ij} \sim Multinomial(n_{j},\pi _{j}(x_{ik}))$, whose probability mass function is given by:

2 $$\begin{aligned} f(y_{i1},y_{i2},y_{i3})=\frac{n!}{(\sum y_{i1})! .(\sum y_{i2})!.(1-\sum \nolimits _{j=1}^{2}\sum y_{ij})!}\pi _{1}(x_{ik})^{\sum y_{i1}}.\pi _{2}(x_{ik})^{\sum y_{i2}} . \pi _{3}(x_{ik})^{(1-\sum \nolimits _{j=1}^{2}\sum y_{ij})}. \end{aligned}$$

Therefore, the survey weighted multinomial logit model is given by:

3 $$\begin{aligned} ln \left[ \frac{\pi _{j}(x_{ik})}{\pi _{3}(x_{ik})}\right] =\sum \limits _{i=1}^{n_{j}}\sum \limits _{k=0}^{p}\beta _{jk}x_{ik}w_i,\quad j=1,2;\quad i=1,2,3,...,n_{j},\quad x_{i0} = 1, \end{aligned}$$

where the 3rd category of birth weight outcome is taken as a reference in the model. The maximum likelihood estimation (MLE) method was used to estimate the regression parameters, along with the jackknife technique for computing the survey weighted standard standard errors of the estimates [23]. The MLE estimates are found by taking the product of the probabilities in Eq. (1) for individual children and then taking the logarithm of the result, from which the partial derivatives with respect to model parameters are obtained. Then, the MLE solutions for the model coefficients are found by solving for the coefficients, when the derivatives of the log-likelihood are equated to zero. This is done with the aid of numerical methods, because the model is not linear and the equations arising from derivatives of the log-likelihood function are not in closed form [22]. The expontiated MLE estimate, $exp(\hat{\beta }_{jk})$ is interpreted as the ratio of odds of having birth weight outcome in *j*-th category relative to 3rd category, when comparing one level of a covariate *X* to the other.

We used Akaike Information Criterion (AIC) to select the best model. The initial model, i.e. Model 1 included all the available covariates in the data set that were described in first paragraph. Then, the second model, i.e. Model 2, excluded the covariates whose MLE estimates had the largest *p*-values in Model 1. Finally, the third model, Model 3 excluded covariates that had MLE estimates with large *p*-values in Model 2. In each case, the AIC value was computed, and later compared with the other values across the three models. The ultimate best model was the one that had lowest AIC among the three.

Now, to identify the outlying clusters to the fitted multinomial logit model, we first computed model residuals at individual level of the data. A Pearson’s residual for multinomial logit model (3) [17] is given by:

4 $$\begin{aligned} r_{i}=\frac{e_{i}}{s(e_{i})}= \frac{y_{ij}-n_{j}\hat{\pi }_{j}(x_{ik})}{\sqrt{n_{j}\hat{\pi }_{j}(x_{ik})}}=\frac{y_{ij}-n_{j}\frac{exp(\hat{\beta }_{jk}x_{ik}w_i)}{\sum \nolimits _{s=1}^{j}\sum \nolimits _{k=0}^{p}exp(\hat{\beta }_{jk}x_{ik}w_i)}}{\sqrt{n_{j}\frac{exp(\hat{\beta }_{jk}x_{ik}w_i)}{\sum \nolimits _{s=1}^{j}\sum \nolimits _{k=0}^{p}exp(\hat{\beta }_{jk}x_{ik}w_i)}}}, \end{aligned}$$

where $i = 1,2,...,n_j$, $j=1,2,3$, and $x_{i0}=1$. The residual (4) assesses univariate outliers to model (3). More generally, the sum of squared Peasron’s residual (4) over all levels of *j* is used as a Chi-squared goodness-of-fit statistic for the model. The univariate outliers were also assessed using the deviance residual [17] given by:

5 $$\begin{aligned} d^{2}_{i}=2\sum \limits _{j=1}^{3}\left[ y_{ij}log\frac{y_{ij}}{n_{j}\hat{\pi }_{j}(x_{ik})}\right] =2\sum \limits _{j=1}^{3}\left[ y_{ij}log\frac{y_{ij}}{n_{j}\frac{exp(\hat{\beta }_{jk}x_{ik}w_i)}{\sum \nolimits _{s=1}^{j}\sum \nolimits _{k=0}^{p}exp(\hat{\beta }_{jk}x_{ik}w_i)}}\right] . \end{aligned}$$

The two methods given in Eqs. (4) and (5) yield similar results. They both have approximate normal distibution, and hence can report outliers to the model at a cutoff of $\pm 2.5$ [25]. To identify grouped outliers for multinomial logit model (3) at cluster level, the method of local mean deviance suggested by Jennings [26] was used by averaging the deviance residuals (5) over each cluster, given by:

6 $$\begin{aligned} D_{c}=\overline{d^{2}_{ic}}= \frac{\sum \nolimits _{i=1}^{n_{c}} {d^{2}_{i}(\hat{\pi }_{j}(x_{ik}),n_{j})}}{n_{c}-1}, \end{aligned}$$

where $d^{2}_{i}(.)$ is the deviance residual given in Eq. (5), $c = 1,2, ..., 850$ is the cluster identification number and $n_{c}$ is *c*-th cluster sample size. The value of $D_c$ that is very large compared to others will indicate a cluster that has a different pattern of child birth weight outcomes compared to the others. This was shown by plotting the values of the residual (6) against cluster identification numbers.

To assess influence of individual observations on estimated regression coefficients to model (3), a generalised Cook’s distance for model (3) [25, 27] was used, which is given by:

7 $$\begin{aligned} \Delta \hat{\beta }_{i} = \frac{r_{i}^{2}h_{ii}}{(1-h_{ii})^{2}} \end{aligned}$$

where $r_{i}$ is the Pearson’s redidual (4), $h_{ii}$ is the *i*-th diagonal element of the leverage matrix $H=V^{1/2}\mathbf {X}(\mathbf {X}^{T}V\mathbf {X})^{-1}\mathbf {X}^{T}V^{1/2}$, with *V* as estimated variance of $y_{ij}$. Usually, an observation that has larger value than 1 is considered influential to the regression estimates [25]. Similarly, grouped influence was estimated using the method of [26] through averaging the Cook’s distance (7) over each cluster, and graphically assessing influential clusters to the MLE estimates in the model. We used cluster-by-cluster comparisons of the computed mean residual values to identify the unusual clusters, without necessarily having a specific cutoff [9, 16].

## Results

The results in Table 1 showed that majority of the children in Malawi were born with normal birth weight, followed by low birth weight and high birth weight in that order. The cases of low birth weight were concentrated in children from rural areas, poor households, mothers with no education, first birth order children, smoking mothers during pregnancy, twin births, non-caesarian births, and in mothers aged below 20 years. While cases of high birth weight were concentrated in children from rich households, urban locations, mothers with secondary or higher education, caesarian births, and mothers aged above 20 years. The chi-square test showed a significant association between each of these variables and birth weight (*p*-value < 0.001).

**Table 1:** Distribution of child birth weight outcomes over socio-demographic characteristics of the children, 2015-16 MDHS (*n* = 11,680)

Characteristic	Total (%)	<2,500g (%)	2,500-4,000g (%)	>4,000g (%)	*X*^2^*p*-value
Overall sample	11,680 (100)	1,940 (16.61)	8,428 (72.16)	1,312 (11.23)	
Mother’s age at birth					
<20	962 (8.05)	223 (21.86)	653 (68.98)	86 (9.16)	
20-34	8,478 (74.11)	1,338 (14.88)	6,211 (75.01)	929 (10.10)	<0.0001
35-49	2,240 (17.84)	379 (16.37)	1,564 (70.38)	297 (13.25)	
Birth order					
1	2,825 (26.70)	570 (18.64)	2,021 (73.48)	234 (7.88)	
2-3	4,432 (40.44)	675 (13.93)	3,258 (75.48)	499 (10.59)	<0.0001
4-5	2,779 (22.06)	430 (15.34)	2,006 (72.69)	343 (11.97)	
6+	1,644 (10.80)	265 (15.87)	1,143 (69.67)	236 (14.46)	
Mother’s education					
No education	1,206 (7.68)	229 (18.47)	821 (69.23)	156 (12.31)	
Primary	7,529 (56.73)	1,300 (17.01)	5,333 (71.20)	896 (11.79)	
Secondary	2,703 (31.45)	389 (13.60)	2,066 (77.66)	248 (8.74)	<0.0001
Higher	242 (4.15)	22 (8.82)	208 (86.22)	12 (4.97)	
Wealth index					
Poor	4, 711 (7.68)	881 (18.47)	3,276 (69.23)	554 (12.31)	
Rich	2,229 (14.23)	375 (17.24)	1,586 (71.49)	268 (11.28)	<0.0001
Richer	4,740 (58.04)	684 (13.97)	3,566 (76.24)	490 (9.79)	
Place of residence					
Urban	2,164 (18.53)	309 (14.28)	1,648 (76.16)	207 (9.57)	
Rural	9,516 (81.47)	1,631 (17.14)	6,780 (71.25)	1,105 (11.61)	<0.001
Mother smokes					
No	11,619 (99.52)	1,930 (15.68)	8,384 (73.74)	1,305 (10.59)	<0.001
Yes	61 (0.48)	10 (22.05)	44 (66.60)	7 (11.35)	<0.001
Twin birth					
No	11,446 (98.15)	1,817 (15.00)	8,328 (74.29)	1,301 (10.71)	
Yes	234 (1.85)	123 (53.14)	100 (42.78)	11 (4.08)	<0.001
ANC visits					
<3	1,389 (11.21)	320 (20.69)	954 (69.25)	133 (10.06)	
3-7	10,084 (86.78)	1600 (15.08)	7,330 (74.21)	1,154 (10.71)	<0.001
>7	207 (2.02)	38 (15.22)	144 (76.50)	25 (8.29)	
Child’s Sex					
Female	5,932 (51.38)	836 (13.38)	4,320 (74.13)	776 (12.49)	
Male	5,748 (48.62)	1,104 (18.17)	2,108 (73.25)	536 (8.58)	<0.001
Caesarean birth					
No	10,817 (90.60)	1,804 (53.78)	7,862 (74.43)	1,151 (9.79)	
Yes	827 (9.40)	132 (15.73)	538 (73.70)	157 (15.70)	<0.001

The results in Table 2 showed that Model 2, that excluded effects of place of residence and mother’s smoking status in predicting birth weight, fitted the data well, as it had the lowest AIC value. The goodness-of-fit test results showed that birth weight variable followed multinomial probability distribution (*p*-value = 0.764). The MLE estimates in Table 2 showed that chances of a child being born with high birth weight relative to low birth weight were more than double in caesarean section births compared to regular delivery. Further, male children had significantly lower relative chances of being born with normal or high birth weight compared to female children. In addition, the relative chances of normal or high birth weight were more than 40% in children whose mothers attended 3 or more antenatal care clinics compared to less than 3. It was also observed that twin births had significantly reduced relative chances of being born with normal or high birth weight compared with singleton births.

Furthermore, children of birth order 2 and above had significantly increased relative chances of being born with normal birth or high birth weight compared to birth order 1. Essentially, these chances almost doubled for high birth weight from the normal birth weight. Further, the relative chances of being born with normal birth weight were higher in children from mothers with primary education and above compared to no education. Similar trend was observed for high birth weight versus mother’s education, although the estimates were not statistically significant. The results also showed that children from richer households had 26% higher relative chances of being born with normal birth weight compared to children from poor household. Finally, there was no significant difference in chances of being born with normal or high birth weight when comparing different levels of mother’s age.

**Table 2:** Effect of child characteristic on birth weight outcomes upon fitting multinomial logit model to child data, 2015-16 MDHS

	Model 1		Model 2		Model 3	
Variable	RRR-NBW (*p*-value)	RRR-HBW (*p*-value)	RRR-NBW (*p*-value)	RRR-HBW (*p*-value)	RRR-NBW (*p*-value)	RRR-HBW (*p*-value)
Mother’s age’						
<20*						
20−34	1.14 (0.261)	1.00(0.987)	1.14 (0.271)	1.00 (0.984)		
35−49	0.83 (0.24)	0.84 (0.504)	0.83 (0.249)	0.84 (0.503)		
Birth order						
1*						
2-3	1.51 (<0.001)	2.00 (<0.001)	1.51 (<0.001)	2.00 (<0.001)	1.54 (<0.001)	1.98 (<0.001)
4-5	1.64 (<0.001)	2.37 (<0.001)	1.64 (<0.001)	2.36 (<0.001)	1.55 (<0.001)	2.23 (<0.001)
6+	2.01 (<0.001)	3.36 (<0.001)	2.00 (<0.001)	3.39 (<0.001)	1.63 (<0.001)	2.94 (<0.001)
Mother’s educ						
No educ*						
Primary	1.11 (0.353)	1.16 (0.317)	1.11 (0.332)	1.16 (0.319)	1.13 (0.251)	1.17 (0.276)
Secondary	1.48 (0.004)	1.13 (0.528)	1.50 (0.003)	1.12 (0.549)	1.53 (0.001)	1.13 (0.501)
Higher	2.62 (<0.001)	0.93 (0.873)	2.71 (0.001)	0.92 (0.847)	2.71 (<0.001)	0.92 (0.909)
Wealth index						
Poor*						
Rich	1.05 (0.610)	0.99 (0.950)	1.05 (0.563)	0.99 (0.929)	1.05 (0.567)	0.99 (0.920)
Richer	1.23 (0.026)	1.09 (0.857)	1.26 (0.005)	1.05 (0.629)	1.24 (0.008)	1.04 (0.692)
Residence						
Urban*						
Rural	0.96 (0.658)	1.07 (0.592)				
Twin birth						
No*						
Yes	0.14 (<0.001)	0.07 (<0.001)	0.14 (<0.001)	0.07 (<0.001)	0.14 (<0.001)	0.07 (<0.001)
Mother smokes						
No*						
Yes	0.71 (0.630)	0.80 (0.822)				
ANC visits						
<3*						
3-7	1.44 (<0.001)	1.48 (0.009)	1.44(<0.001)	1.48 (0.009)	1.44 (<0.001)	1.48 (0.009)
>7	1.53 (0.121)	1.24 (0.599)	1.51 (0.125)	1.24 (0.591)	1.48 (0.146)	1.22 (0.613)
Child’s Sex						
Female*						
Male	0.73 (<0.001)	0.51 (<0.001)	0.72 (<0.001)	0.51 (<0.001)	0.72(<0.001)	0.51 (<0.001)
Caesarean						
No*						
Yes	0.88 (0.305)	2.32 (<0.001)	0.88 (0.314)	2.31 (<0.001)	0.87 (0.294)	2.30 (<0.001)
AIC	18,085.51		18,082.75		18,085.37	

RRR = relative risk ratio; age’= age at child birth; * means reference level

**Fig. 1:**
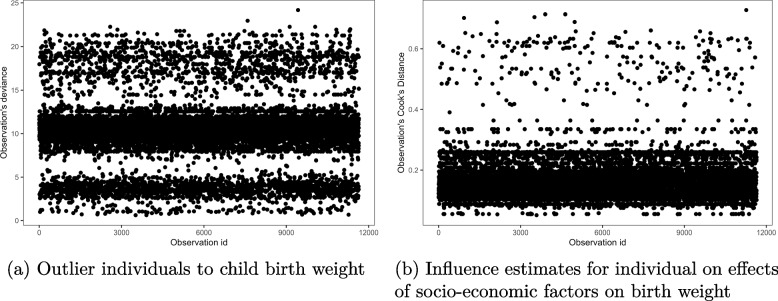
Individual observations outlier and influence estimates from the multinomial logit model for birth weight outcomes, 2015-16 MDHS data. Source: Researcher

The univariate deviance residual estimates in Fig. 1(a) showed that the estimated birth weights were close to each other, with none outlying in the fitted multinomial logit model. Similarly, the Cook’s distance estimates in Fig. 1(b) were all less than 1, indicating that none of the observations had overly individual influence on the parameter estimates in the model. However, most values of the Cook’s distance were concentrated towards zero, with few more hanging isolated towards one. This indicated that the observed lack of influence was not uniform among the observations, some still portrayed a deviation in the fit.

**Fig. 2:**
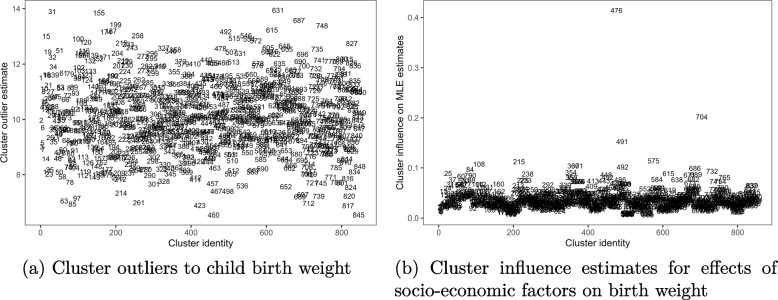
Cluster outlier and influence estimates based on multinomial logit model for birth weight outcomes, 2015-16 MDHS data. Source: Researcher

Now, considering the clustered mean deviance residuals in Fig. 2(a), it is shown that none of the clustered observations deviated from the fitted multimonial model. This agreed with univariate estimates in Fig. 1(a). While the cluster averaged Cook’s distances in Fig. 2(b) showed that the observations for children in cluster 476, that is in rural part of Nsanje district and 704 from rural part of Thyolo district had joint excessive influence on the regression model coefficients estimates. This implies that removing each of the two clusters from the analysis could impact on the MLE estimates substantially than the rest clusters would.

## Discussion

This study applied univariate model diagnostic statistics to analyse unusual child birth weight outcomes at community level, upon fitting a multinomial logit model to data. The findngs showed that none of the observations deviated from the fitted model both at individual and cluster levels. This reflects the fact that majority of the babies that were studied were born with normal birth weight and very few had low or high birth weight. However, it was observed that two clusters had overly influence on estimated effects of socio-demographic factors on birth weight. Influence of a subject to the fitted model reflects impact the subject causes to the estimates of the regression coefficients, and it is the product of outlierness of that subject on the fitted line or curve and its leverage on the fitted response [9, 16]. The influence value signals the effect that inclusion or exclusion of the subject in the analysis would cause to the model estimates. Obviously, inclusion of the two clusters in the analysis biased the estimates of the effects of the various covariates on child birth weight. The back-inspection of the data showed that the two influential clusters were from rural parts in two districts of Thyolo and Nsanje in Southern Malawi and had all children with normal or high birth weight. Further, it was observed that all the sampled births in the two influential clusters were non-caesarian, singleton, and of birth order 1. It was indeed unusual for the two rural-based clusters to have most children with normal-to-high birth weight, since it is low birth weight that is prevalent in rural areas, in non-caesarian births, and birth order of 1 [20].

The model estimates showed that birth order of 2 and above had increased chances for a baby to be born with normal and high birth weight relaitve to low birth weight compared to birth order 1. This is a common observation in most studies, but there has been little scientific explanation in research attached to it [20]. The results also showed that caesarian births had high chances of being high birth weight relative to low birth weight compared with regular births. While chances were low for male babies to be born with normal or high birth weight relative to low birth weight compared to female babies. In addition, twin births had reduced chances of being born with normal or high birth weight relative to low birth weight compared to singleton briths. These results are consistent with findings from previous studies and have relevant biological reasons attached to them [19, 28]. Furthermore, it was observed that antenatal care clinic visits of above 3 had increaded chances for a baby to be born with normal birth weight relative to low birth weight compared to less than 3 clinic visits. These benefits of antenatal clinic visits have also been reported in other studies and relate to inculcating knowledge to the expectant mother on nutritional requirements during preganacy that relate with child birth weight [28].

## Conclusion

This study investigated unusual communities to child birth weight in Malawi using the multinomial regression model diagnostics. The study has identified two clusters from rural parts of Southern Malawi that had excessive influence on effects of various socio-demographic factors on birth weight, which biased the estimates. The two rural clusters had children with normal or high birth weight, with no cases of low birth weight, which was not common in rural locations of the study population. We recommend a follow-up qualitative study to investigate child healthcare practices that mothers of the area follow, which may be replicated in the other rural parts of the country. These findings imply that some classical statistical diagnostic methods can be utilised further to understand the outlying patterns of children’s health outcomes at community level.

## Data Availability

The 2015-16 MDHS data is publicly and freely available for users at https://dhsprogram.com/data/new-user-registration.cfm.
